# Application of dynamic modeling for survival estimation in advanced renal cell carcinoma

**DOI:** 10.1371/journal.pone.0203406

**Published:** 2018-08-30

**Authors:** Baris Deniz, Arman Altincatal, Apoorva Ambavane, Sumati Rao, Justin Doan, Bill Malcolm, M. Dror Michaelson, Shuo Yang

**Affiliations:** 1 Evidera Inc., Bethesda, Maryland, United States of America; 2 Evidera Inc., Waltham, Massachusetts, United States of America; 3 Evidera Inc., London, United Kingdom; 4 Bristol-Myers Squibb, Princeton, New Jersey, United States of America; 5 Massachusetts General Hospital, Boston, Massachusetts, United States of America; University of Washington, UNITED STATES

## Abstract

**Objective:**

In oncology, extrapolation of clinical outcomes beyond trial duration is traditionally achieved by parametric survival analysis using population-level outcomes. This approach may not fully capture the benefit/risk profile of immunotherapies due to their unique mechanisms of action. We evaluated an alternative approach—dynamic modeling—to predict outcomes in patients with advanced renal cell carcinoma. We compared standard parametric fitting and dynamic modeling for survival estimation of nivolumab and everolimus using data from the phase III CheckMate 025 study.

**Methods:**

We developed two statistical approaches to predict longer-term outcomes (progression, treatment discontinuation, and survival) for nivolumab and everolimus, then compared these predictions against follow-up clinical trial data to assess their proximity to observed outcomes. For the parametric survival analyses, we selected a probability distribution based on its fit to observed population-level outcomes at 14-month minimum follow-up and used it to predict longer-term outcomes. For dynamic modeling, we used a multivariate Cox regression based on patient-level data, which included risk scores, and probability and duration of response as predictors of longer-term outcomes. Both sets of predictions were compared against trial data with 26- and 38-month minimum follow-up.

**Results:**

Both statistical approaches led to comparable fits to observed trial data for median progression, discontinuation, and survival. However, beyond the trial duration, mean survival predictions differed substantially between methods for nivolumab (30.8 and 51.5 months), but not everolimus (27.2 and 29.8 months). Longer-term follow-up data from CheckMate 025 and phase I/II studies resembled dynamic model predictions for nivolumab.

**Conclusions:**

Dynamic modeling can be a good alternative to parametric survival fitting for immunotherapies because it may help better capture the longer-term benefit/risk profile and support health-economic evaluations of immunotherapies.

## Introduction

Immune checkpoint antibodies—a relatively new class of oncology treatments—act by blocking inhibitory checkpoints, thus restoring function to the immune system, which can then attack the tumor [1–3]. Immune checkpoint antibodies currently available include programmed death-1 (PD-1) blocking antibodies, such as nivolumab [4] and pembrolizumab [5]; PD-1 ligand 1 (PD-L1) blocking antibodies, such as atezolizumab [6] and avelumab [7]; and cytotoxic T-lymphocyte antigen 4 blocking antibodies, such as ipilimumab [8]. Such immunotherapies have demonstrated beneficial effects on objective response rates, progression-free survival (PFS), and overall survival (OS) in various cancers [9–15].

Outcomes such as treatment response, PFS, and OS are key endpoints to ascertain the clinical and economic benefits of oncology treatments and are used in health economic evaluations [16–19]. Estimating treatment duration, PFS, and OS with immunotherapies over patient lifetimes is critical for understanding their overall value. This typically requires extrapolating clinical trial findings over patient lifetimes, and for this, parametric survival analyses traditionally are used [20]. However, there are several reasons why this approach may not be suitable for immunotherapies. First, survival, treatment duration, and progression risk are heterogeneous—some patients have a strong antitumor response, while others do not. Therefore, using a weighted average of OS for responders and nonresponders using a single parametric fit may not adequately capture the therapeutic value. Second, the response from immunotherapy tends to be highly durable, resulting in long-term efficacy for some patients and hence, a plateau in survival curves [9,21]. Third, immunotherapy can result in nonstandard survival curves with inflection points showing decelerations of hazards [13,22], which cannot be accounted for using single parametric distributions. Thus, to fully assess the potential value of immunotherapies, alternative approaches may have to be considered—e.g., piecewise fitting, landmark analysis, pre- versus post-progression survival, parametric mixture models, spline-based models, and dynamic modeling [23]—as the approach used is likely to affect the value assessment substantially, especially the long-term predictions with immunotherapies.

We conducted a case study that illustrates clinical outcomes using the traditional standard parametric survival analysis approach versus an alternative approach (dynamic modeling) to understand differences in extrapolation results. Dynamic modeling was selected because it allows real-time prediction of events using time-dependent factors (such as response). For this study, we modeled outcomes (progression, treatment discontinuation, and survival) and compared them against clinical data from patients who received nivolumab or everolimus for treatment of advanced renal cell carcinoma (RCC) in a recent phase III study [12]. We also compared the predicted outcomes using both statistical models against extended trial follow-up from the same phase III study.

## Methods

### Simulation model

**Fig 1:**
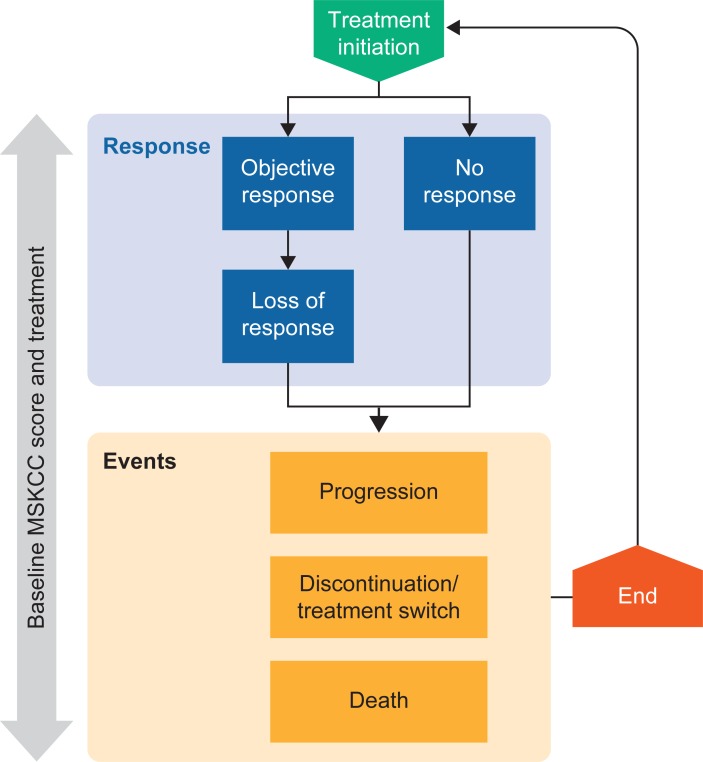
Simulation model structure. Note: Impact of response achievement and loss of response on progression, discontinuation, and death is only considered for the dynamic modeling approach.

A simulation model (Fig 1) was developed in Microsoft Excel using the discretely integrated condition event (DICE) method, which served as the framework that accommodated both the standard parametric approach and dynamic modeling as the underlying predictive equations within the same model structure and set of assumptions [24].

The model predicted health outcomes over patient lifetimes (25 years), more specifically times to treatment discontinuation (TTD), progression (TTP), and death (TTDeath) for patients with advanced or metastatic clear-cell RCC who had received one or two prior antiangiogenic therapy regimens. In addition to TTP, TTD, and TTDeath, the dynamic modeling also included estimations of times to response and to loss of response for patients with an objective response, as these measures were identified as time-dependent parameters that influence long-term outcomes.

### Data source

Data for analysis were taken from the randomized, phase III CheckMate 025 study [12], in which 821 patients with advanced or metastatic clear-cell RCC who had previously received one or two antiangiogenic regimens were randomized to nivolumab (3 mg/kg intravenously every 2 weeks) or to everolimus (10 mg orally once daily). Data were from a June 2015 cutoff date (minimum follow-up, 14 months). The modeled outcomes were compared with the observed Kaplan–Meier (KM) curves from CheckMate 025 for TTD, TTP, and TTDeath [12,25]. KM curves were also stratified to ascertain the impacts of treatment effect, objective response (complete or partial response), and Memorial Sloan Kettering Cancer Center (MSKCC) risk group [26] on clinical outcomes.

### Standard parametric survival analysis

Parametric distributions—Weibull, exponential, log-normal, log-logistic, Gompertz, and generalized gamma [27,28]—were fitted to the observed trial data. We tested the fits of distributions based on [27] graphical assessment (visual inspection, parametric plots, and probability plots), goodness-of-fit measures (Akaike’s information criterion and Bayesian information criterion (AIC/BIC) [20]), and clinical plausibility of fits.

### Dynamic modeling

Dynamic modeling allows real-time prediction of time-to-events using longitudinal markers as time-dependent covariates in a Cox regression model [23,29]. Of the available projection techniques, dynamic modeling [23] was selected to enable 1) incorporation of the impact of important prognostic factors (MSKCC risk group); 2) incorporation of the impact of objective response achievement (partial response or better according to Response Evaluation Criteria In Solid Tumors [RECIST] 1.1 criteria) and duration of response as time-dependent predictors [30], as this is an important outcome of the mechanism of action of immunotherapies [31,32]; and 3) accounting for the unique shape of the KM curves, which can show deceleration of hazards at single or multiple inflection points for immunotherapies.

To account for these features, we conducted a Cox regression analysis, as Cox models do not rely on any specific assumption on the parametric shape of the hazard function. In this analysis, the objective response is treated as a time-dependent indicator, which helps to avoid any bias that may occur if objective response was treated in the same way as a baseline predictor, as it fails to account for the fact that patients had to survive long enough to achieve an objective response.

Cox regression analysis was conducted to determine the impact of objective response and MSKCC risk on TTD, TTP, and TTDeath in the treatment arms. Analyses were conducted using univariate and multivariate approaches. In the univariate approach, each predictor was tested alone in the regression model to assess univariate effects on the outcome. Subsequently, a multivariate model was implemented where treatment arm, objective response, and MSKCC risk group were included. Additionally, a proportional hazards assumption for the Cox regression models was assessed. As the proportional hazard assumption was violated, a piecewise hazard ratio (HR) model was implemented to assess the inflection point at which the hazards differed. Based on this assessment, a model using a time-dependent treatment HR of 0 to 3 and >3 months was used. Since the proportional hazards assumption was not met, we used time-dependent HRs to allow application of Cox regression analysis to the reference arm (comprising patients receiving everolimus with no response and poor MSKCC risk group).

## Results

### Study outcomes

CheckMate 025 showed that patients receiving nivolumab versus everolimus had improvements in objective response rates (25% vs 5%; odds ratio 5.98; 95% confidence interval [CI] 3.68–9.72; *P*<0.001) and median OS (25.0 vs 19.6 months; HR for death 0.73, 98.5% CI, 0.57–0.93; *P* = 0.002), but similar median PFS (4.6 vs 4.4 months; HR 0.88, 95% CI, 0.75–1.03; *P* = 0.11) [12]. Worsening MSKCC risk adversely affected OS in both arms [25] and objective response achievement in the everolimus arm only (Table 1) [25]. OS and objective response were better in the nivolumab versus everolimus arms in all MSKCC risk groups (Table 1) [25].

**Table 1:** CheckMate 025 study results.

	OS, Median (95% CI), Months		Objective Response, % (SE)	
	Nivolumab	Everolimus	Nivolumab	Everolimus
Overall [12]	25.0 (21.8–NE)	19.6 (17.6–23.1)	25 (2)	5 (1)
MSKCC risk^a^ [25]				
Favorable	NE (NE–NE)	NE (24.7–NE)	21 (3)	7 (2)
Intermediate	21.4 (18.3–NE)	17.7 (15.6–19.9)	27 (3)	5 (2)
Poor	18.2 (10.2–26.7)	8.5 (5.2–11.5)	27 (6)	0 (0)

NE, not estimable; SE, standard error.

^a^Based on interactive voice response system (i.e., randomization stratification level assignment).

### Standard parametric survival analysis

Based on AIC/BIC measures, log-cumulative hazard plots, parametric plots, and visual inspection, none of the parametric distributions provided a better fit to the observed trial data. Hence, Weibull distributions were selected to model TTD, TTP, and TTDeath because they provide a good benchmark for comparison as they have recently been used in oncology modeling, especially with immuno-oncology therapies [33–35]. The parameters associated with TTD, TTP, and TTDeath are listed in S1 Table.

### Multivariate Cox regression analysis

TTD, TTP, and TTDeath for the reference arm (everolimus, no response, poor MSKCC risk) were modeled using Weibull distributions; times to response and to loss of response were modeled using log-normal and Gompertz distributions, respectively (S2 Table).

Achievement of response, MSKCC risk group, and treatment were influential predictors for progression, treatment discontinuation, and death (Table 2).

**Table 2:** Multivariate Cox regression analysis–dynamic modeling.

	Progression HR (95% CI)	Discontinuation HR (95% CI)	Death HR (95% CI)
**Response levels (time dependent)**			
Objective response (vs nonresponse)	0.40 (0.30–0.54)***	0.18 (0.12–0.27)***	0.06 (0.02–0.19)***
Post-objective response (vs nonresponse)	NA	1.34 (0.96–1.88)	0.49 (0.30–0.80)*
**MSKCC risk**			
Favorable (vs poor)	0.63 (0.50–0.81)**	0.64 (0.51–0.80)***	0.29 (0.22–0.39)***
Intermediate (vs poor)	0.79 (0.62–0.99)*	0.79 (0.64–0.98)*	0.61 (0.47–0.79)**
**Treatment (time dependent)**^**a**^			
Nivolumab ≤3 months (vs everolimus ≤3 months)	1.19 (0.94–1.50)	0.73 (0.62–0.85)***^a^	0.50 (0.28–0.90)*
Nivolumab >3 months (vs everolimus >3 months)	0.88 (0.70–1.11)	–^**a**^	0.94 (0.76–1.16)

Data are hazard ratio (95% CI).

NA, not applicable.

*P<0.05.

**P<0.001.

***P<0.0001.

^a^Treatment discontinuation outcome did not utilize a time-dependent treatment effect. The treatment comparison reflects nivolumab vs everolimus across the entire follow-up.

It is important to note that there is a correlation between treatment and its duration with response levels achieved. Objective response achievement significantly reduced risk of progression (HR 0.40; 95% CI, 0.30–0.54), treatment discontinuation (HR 0.18; 95% CI, 0.12–0.27), and death (HR 0.06; 95% CI, 0.02–0.19) compared with nonresponse. Loss of response increased risk of discontinuation and death compared with response achievement. However, patients who achieved and then lost response had a significantly lower risk of death (HR 0.49; 95% CI, 0.30–0.80), but similar risk of discontinuation (HR 1.34; 95% CI, 0.96–1.88) than those not achieving response. Favorable and intermediate MSKCC risk significantly reduced risk of progression (HR 0.63; 95% CI, 0.50–0.81 and HR 0.79; 95% CI, 0.62–0.99, respectively), discontinuation (HR 0.64; 95% CI, 0.51–0.80 and HR 0.79; 95% CI, 0.64–0.98), and death (HR 0.29; 95% CI, 0.22–0.39 and HR 0.61; 95% CI, 0.47–0.79) compared with poor MSKCC risk (Table 2).

Compared with everolimus, nivolumab increased the risk of progression within 3 months of follow-up. However, with >3 months of follow-up, nivolumab slightly reduced the risk of progression compared with everolimus (both not significant at 0.05 level) (Table 2). The risk of progression on nivolumab may have been higher in the first 3 months compared with after 3 months because patients on nivolumab can experience a delay in clinical benefit during the early stages of treatment [36]. Patients on nivolumab were significantly less likely to discontinue treatment (HR 0.73; 95% CI, 0.62–0.85; *P*<0.0001) and less likely to die during the first 3 months (HR 0.50; 95% CI, 0.28–0.90; *P* = 0.02) than those on everolimus (Table 2).

### Comparison of dynamic modeling versus standard parametric survival analysis

**Fig 2:**
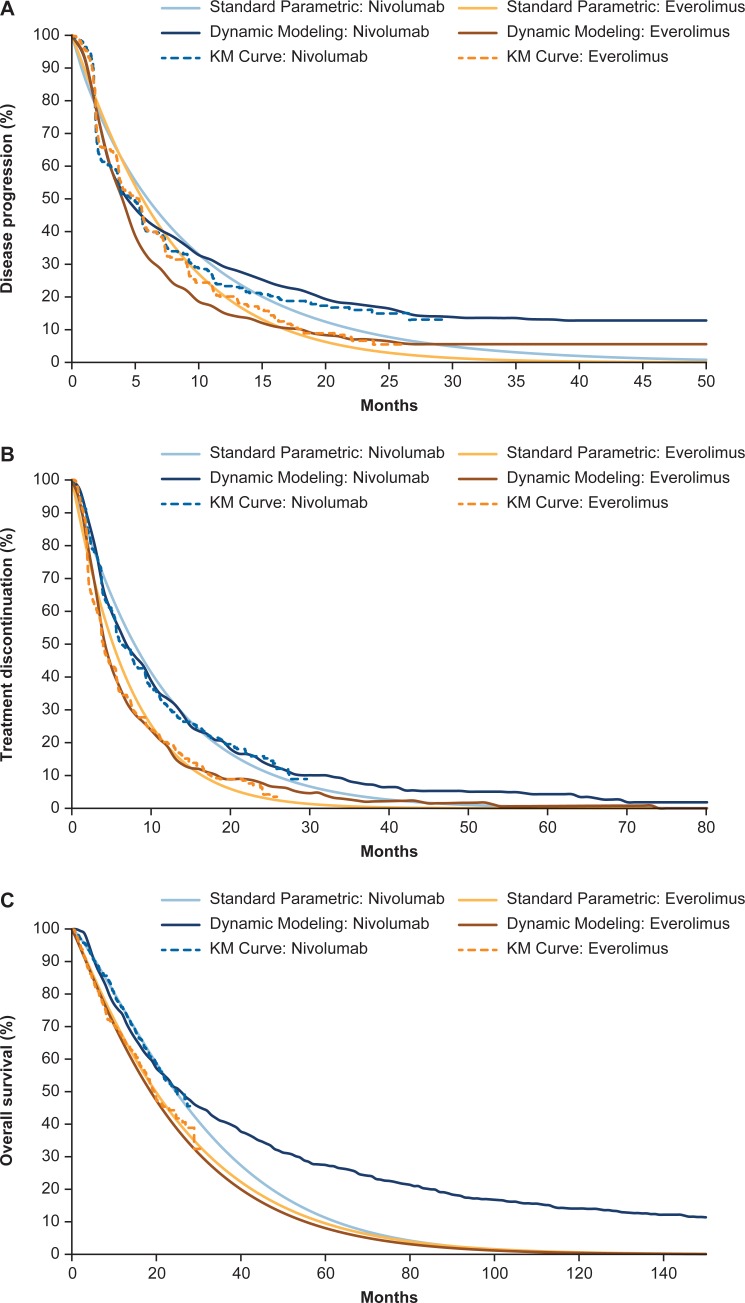
Comparison of KM curves (CheckMate 025 study data), standard parametric analysis, and dynamic modeling curves for (A) progression, (B) treatment discontinuation, and (C) overall survival.

For TTP, dynamic modeling showed a better fit to the observed trial data compared with the standard parametric fit, particularly for nivolumab (Table 3, Fig 2A).

**Table 3:** Descriptive statistics summary for TTD, TTP, and TTDeath (25-Year horizon).

	TTP		TTD		TTDeath	
	Nivolumab	Everolimus	Nivolumab	Everolimus	Nivolumab	Everolimus
**Months, median**						
Trial-reported [12,25]	4.6	4.3	5.4	3.7	25.0	19.6
Standard parametric analysis^a^	5.0	5.0	7.0	5.0	24.0	19.0
Dynamic modeling^a^	4.4	3.6	6.2	4.1	27.0	19.2
**Months, mean**						
Standard parametric analysis^a^	9.8	8.0	11.8	7.8	30.8	27.2
Dynamic modeling^a^	12.8	7.3	10.2	6.0	51.5	29.8

^a^Based on time-to-event summaries.

This is because dynamic modeling accounts for varying HRs across the follow-up (e.g., ≤3 vs >3 months), thus enabling better modeling of the nivolumab treatment effect. The piecewise HR also allows the capture of the higher risk of progression during the first 3 months versus after 3 months. For TTD, dynamic modeling showed a better fit to the observed trial data compared with the standard parametric fit for nivolumab and everolimus (Table 3, Fig 2B). Similarly to TTP, the Cox regression accounted for time-dependent changes in treatment effect. For TTDeath, both standard parametric survival analysis and dynamic modeling compared well versus the observed portion of the trial data (Table 3, Fig 2C).

Beyond the trial duration, mean TTDeath predictions for everolimus were similar with both methods, but differed substantially between the parametric and dynamic methods for nivolumab (30.8 and 51.5 months, respectively) due to the estimated role of key clinical events on TTDeath (Table 3, Fig 2C).

## Discussion

This case study provides a comparison of a standard parametric approach that is commonly used in health economic modeling studies and dynamic modeling for fitting survival curves in patients with advanced RCC. Dynamic modeling showed better or similar fits to the observed trial data than the standard parametric approach for median treatment discontinuation, progression, and OS in both the nivolumab and everolimus arms. This is most likely because dynamic modeling can account for patient characteristics and varying hazards, thus matching the observed trial data to the unique shape of the KM curves using a Cox regression analysis. The extrapolation techniques help quantify the treatment value over patient lifetime, which cannot be estimated with limited follow-up clinical trial data. Predictive models were developed using observed trial data (minimum follow-up 14 months) and validated against extended data cuts (minimum follow-up 26 and 38 months).

Mean OS predictions with dynamic modeling were similar to those obtained with the standard parametric approach for everolimus (29.8 and 27.2 months, respectively), but for nivolumab, there was a large difference between the approaches (51.5 and 30.8 months, respectively). Accuracy and proximity of estimates to the observed mean OS cannot be confirmed until long-term trial data beyond 5 years become available. However, using data from a later data cutoff date from CheckMate 025 (May 2016 data cutoff, minimum follow-up 26 months, and June 2017 data cutoff, minimum follow-up ~38 months) [37,38], the predicted survival curve using the dynamic approach produced a fit to the observed KM curve that was better than the standard parametric approach curve (S1 Fig). Longer follow-up of patients with advanced RCC treated with nivolumab are available from phase I (N = 34) and II (N = 167) studies [39]. The phase I study reported 3- and 5-year OS rates of 41% and 34%, respectively (minimum follow-up, 50.5 months); the phase II study reported 3-year OS of 35% (minimum follow-up, 38 months) [39]; and CheckMate 025 reported 3-year OS rates of 39% [38]. Dynamic modeling of CheckMate 025 data predicted 3- and 5-year OS rates of 41% and 28%, similar to the phase I and II data and the later data cut from CheckMate 025 [38]. In contrast, the standard parametric approach predicted 3- and 5-year OS rates of 30% and 10%, and although the 3-year estimate was similar to the trial data, the 5-year estimate was much lower. These longer-term follow-up data indicate that dynamic modeling may be more suitable than the standard parametric approach for nivolumab in advanced RCC. This further supports the need for alternative modeling methods for immunotherapies, to account for their unique mode of action. In the context of immunotherapies, response levels and durations seem to a play very important role in predicting longer-term outcomes.

As the accuracy of survival curve estimates can vastly affect subsequent cost-effectiveness analysis results, the choice of survival modeling method is very important. In a recent cost-effectiveness evaluation of nivolumab as a second-line treatment for advanced RCC, Wan et al. [40] used a partitioned survival model to estimate costs, life-years, and quality-adjusted life-years. The authors used data from CheckMate 025 [12], but did not have access to individual patient data. They therefore used a method published by Hoyle and Henley [41] to estimate the underlying individual patient data from the numbers at risk from the overall population KM graphs. The survival curves were then fitted using a Weibull distribution. This resulted in mean PFS and OS estimates of 9.3 and 30.9 months, respectively, for nivolumab and 7.3 and 27.0 months, respectively, for everolimus [40]. These values are all similar to our standard parametric results (Table 3), with the largest difference being 0.7 months (for everolimus PFS). However, the OS estimate for nivolumab was substantially shorter than our dynamic estimate (30.9 vs 51.5 months) [40]. While the long-term predictions from Wan et al. cannot be validated against the longer follow-up data presented here, dynamic modeling estimates compare well against the phase I/II trial data.

Different modeling approaches for the extrapolation of survival curves, performed with a view to performing cost-effectiveness analyses related to immunotherapies for indications other than advanced RCC, were compared earlier by Bohensky et al. [42]. In that study, the authors compared Weibull, log-logistic, and a Weibull mixture cure model for estimating survival in previously untreated patients with advanced melanoma treated with nivolumab versus ipilimumab. Estimated 3-year survival results from the three models varied widely. Bohensky et al. concluded that the choice of model would have a substantial impact on predicted effectiveness and cost-effectiveness, hence they recommended that different models should be considered in sensitivity analyses. While this point can be argued more generally for studies that project short-term outcomes into the future, it is particularly important for immunotherapies due to their unique mechanism of action: immunotherapies tend to have higher and more durable response rates than traditional chemotherapies or targeted therapies, which is likely to impact treatment discontinuation, progression, and survival. The observed KM curves from trials also suggest that immunotherapies may be associated with varying hazards over time, which show signs of deceleration at some points that requires careful exploration of inflection points and adjustments for changes in hazard before/after these time points.

Various statistical techniques—piecewise fitting, landmark analysis, pre- versus post-progression survival, parametric mixture models, and spline-based models [23]—are considered for extrapolation of clinical outcomes. These methods may address one or multiple nuances of immunotherapy mechanism of action. Due to its flexibility and adaptability, dynamic modeling is a promising alternative methodology that can address most of the intricacies immunotherapies introduce; hence, it was compared with standard parametric survival analysis. The comparability of alternative extrapolation techniques can be considered for future research. Dynamic modeling allows the incorporation of the impact of patient heterogeneity; varying hazards over time, such as deceleration of hazards seen after 3 months in this example; and interdependencies of clinical outcomes. For instance, in our example, the separation of KM curves for responders and nonresponders for treatment discontinuation, progression, and survival suggest—not surprisingly—that these subgroups have varying risks (S2 Fig). Furthermore, achieving response is positively correlated with survival despite losing response later, which can be captured with dynamic modeling using response as a time-dependent predictor in estimations.

In this case example, the dynamic model incorporated MSKCC risk, objective response achievement and duration of response (both time-dependent predictors), and deceleration of hazards for patients with advanced RCC. The objective of our model was to provide inputs relating to TTP, TTD, and TTDeath for use in a health economic analysis to assess the economic consequences associated with the management of advanced RCC using different treatment sequences, the results of which will be presented elsewhere.

### Limitations

One limitation of our dynamic model is in not accounting for all patient characteristics. Only MSKCC risk group—which incorporates time from diagnosis to treatment, hemoglobin levels, calcium levels, lactate dehydrogenase levels, and Karnofsky performance status—was considered, but other patient characteristics may also impact outcomes. Furthermore, MSKCC risk group was not modeled dynamically; only baseline MSKCC risk score was considered, rather than including this as a time-dependent factor. Finally, MSKCC risk group has only been validated in targeted therapies, not in checkpoint inhibitors.

For the standard parametric approach, we tested various distributions, but as none provided a particularly good fit to the trial data, Weibull distributions were used, as these have been used in various economic analyses of targeted therapies in metastatic RCC [33–35]. We only compared the dynamic modeling approach versus the Weibull parametric approach, and did not compare against other distributions.

We cannot be sure that inflection points in the survival curves are true effects of nivolumab or simply aberrations. Also, long-term extrapolations of the dynamic model are suggestive of cure potential with nivolumab for advanced RCC. However, long-term follow-up data are not available to validate the curative potential. This also makes it impossible to know whether the longer OS estimate for nivolumab using the dynamic approach is more—or less—realistic than the one obtained with the standard parametric approach. However, the good match of the dynamic results with data from a later data cutoff point (3 years) from CheckMate 025 [37,38] and longer-term follow-up data from phase I and II studies [39] are encouraging in this regard.

Lastly, we chose to study the dynamic approach, and did not compare this with possible other approaches, such as piecewise fitting, landmark analysis, pre- versus post-progression survival, parametric mixture models, and spline-based models. Further studies could be undertaken to examine whether these approaches might be suitable for predicting survival curves in patients taking immunotherapies.

## Conclusions

Based on the current study, different statistical approaches can predict considerably different potential long-term benefits for immunotherapies, particularly in terms of TTDeath, which could have an important impact on predicted clinical and economic values of the therapy. Dynamic modeling—which can account for patient heterogeneity and time-dependent disease milestones implemented in flexible platforms (e.g., DICE)—could be a good alternative to survival partition models for immunotherapies.

## Supporting information

S1 Table Parameters for TTD, TTP, and TTDeath–Standard parametric analysis. (DOCX)

S2 Table Parameters for responders and for patients in the reference Arm^a^–Dynamic modeling. (DOCX)

S1 Fig Overall survival: dynamic model prediction versus observed data–June 2015 cutoff (minimum 14 months follow-up)[1] May 2016 (minimum 26 months follow-up), and July 2017 (minimum 28 months follow-up)[2,3] from CheckMate 025. (DOCX)

S2 Fig KM curves for (A and B) TTP, (C and D) TTD, and (E and F) TTDeath, stratified by (A, C, E) objective response or (B, D, F) lack of objective response.
(DOCX)
