# Supplementary material for: Application of dynamic modeling for survival estimation in advanced renal cell carcinoma
Source: PLoS One. 2018 Aug 30;13(8):e0203406. doi: 10.1371/journal.pone.0203406 (PMC6117067; doi:10.1371/journal.pone.0203406)
Supplement: S1 Table — (DOCX) [file pone.0203406.s001.docx]

**S1 Table. Parameters for TTD, TTP, and TTDeath – Standard Parametric Analysis.**

| **Event** | **Distribution** | **Intercept (SE)** | **Scale (SE)** |
| --- | --- | --- | --- |
| TTD – nivolumab | Weibull | 2.4241 (0.0536) | 1.0209 (0.0453) |
| TTD – everolimus | Weibull | 1.9977 (0.0516) | 1.0419 (0.0420) |
| TTP – nivolumab | Weibull | 2.1913 (0.0627) | 0.9130 (0.0410) |
| TTP – everolimus | Weibull | 2.0561 (0.0533) | 1.0832 (0.0471) |
| TTDeath – nivolumab | Weibull | 3.4885 (0.0666) | 1.2880 (0.0871) |
| TTDeath – everolimus | Weibull | 3.3220 (0.0663) | 1.1061 (0.0679) |

SE, standard error; TTD, time to treatment discontinuation; TTP, time to progression; TTDeath, time to death.
