# Supplementary material for: Application of dynamic modeling for survival estimation in advanced renal cell carcinoma
Source: PLoS One. 2018 Aug 30;13(8):e0203406. doi: 10.1371/journal.pone.0203406 (PMC6117067; doi:10.1371/journal.pone.0203406)
Supplement: S2 Table — (DOCX) [file pone.0203406.s002.docx]

**S2 Table. Parameters for Responders and for Patients in the Reference Arm^a^ – Dynamic Modeling.**

| **Event** | **Distribution** | **Intercept (SE)** | **Scale/Gamma (SE)** |
| --- | --- | --- | --- |
| Responders |  |  |  |
| TTR – nivolumab | Log-normal | 1.3261 (0.1383) | 0.64880 (0.0978) |
| TTR – everolimus | Log-normal | 1.2095 (0.0642) | 0.65190 (0.0454) |
| TTLR – nivolumab | Gompertz | 3.0870 (0.4274) | 0.08040 (0.0351) |
| TTLR – everolimus | Gompertz | 2.9128 (0.1803) | 0.07020 (0.0151) |
| Reference arm^a^ |  |  |  |
| TTD | Weibull | 1.5350 (0.1264) | 1.1033 (0.1047) |
| TTP | Weibull | 1.5437 (0.1387) | 1.0743 (0.1094) |
| TTDeath | Weibull | 2.5290 (0.1209) | 1.2129 (0.1460) |

SE, standard error; TTR, time to response; TTLR, time to loss of response; TTD, time to treatment discontinuation; TTP, time to progression; TTDeath, time to death.

^a^Everolimus, no response, poor MSKCC.
