# Supplementary material for: Application of dynamic modeling for survival estimation in advanced renal cell carcinoma
Source: PLoS One. 2018 Aug 30;13(8):e0203406. doi: 10.1371/journal.pone.0203406 (PMC6117067; doi:10.1371/journal.pone.0203406)
Supplement: S1 Fig — (DOCX) [file pone.0203406.s003.docx]

**S1 Fig. Overall survival: dynamic model prediction versus observed data – June 2015 cutoff (minimum 14 months follow-up),[1] May 2016 (minimum 26 months follow-up), and July 2017 (minimum 28 months follow-up)[2,3] from CheckMate 025.**


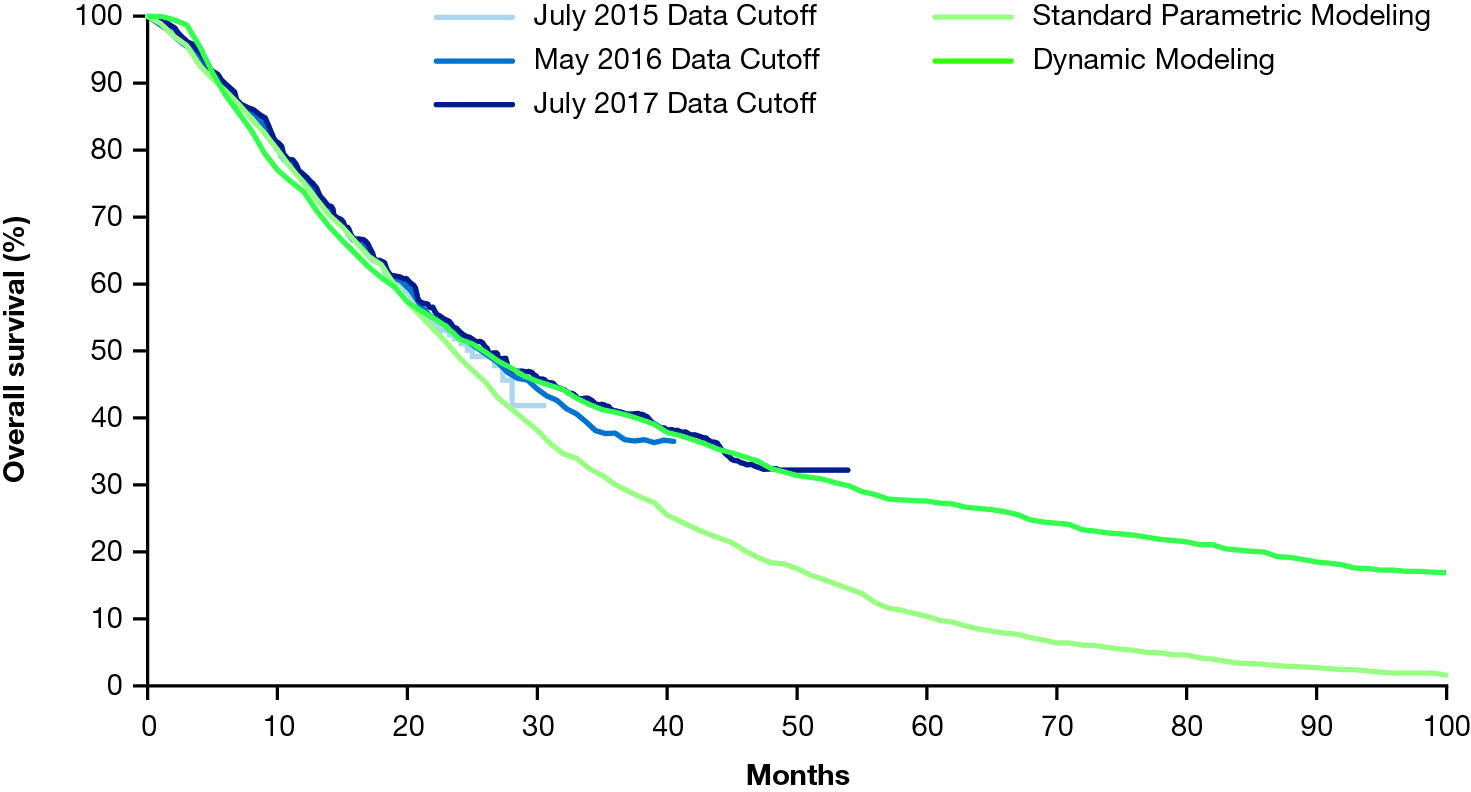


# References

**1.** Motzer RJ, Escudier B, McDermott DF, George S, Hammers HJ, Srinivas S, et al. Nivolumab versus everolimus in advanced renal-cell carcinoma. N Engl J Med. 2015;373(19):1803-13.

**2.** Plimack ER, Motzer RJ, Escudier B, Sharma P, McDermott DF, George S, et al. Two-year efficacy and safety update from the phase III CheckMate 025 study of nivolumab versus everolimus in patients with advanced renal cell carcinoma (aRCC). Poster presented at the 15th International Kidney Cancer Symposium; November 4–5, 2016; Miami, FL, USA.

**3.** Sharma P, Tykodi SS, Escudier B, Carducci M, Oudard S, Hammers HJ, et al. Three-Year Efficacy and Safety Update From the Phase III CheckMate 025 Study of Nivolumab Versus Everolimus in Patients With Advanced Renal Cell Carcinoma. Presented at: 16th International Kidney Cancer Symposium, November 3-4, 2017; Miami, FL, USA.
