# Supplementary material for: Application of dynamic modeling for survival estimation in advanced renal cell carcinoma
Source: PLoS One. 2018 Aug 30;13(8):e0203406. doi: 10.1371/journal.pone.0203406 (PMC6117067; doi:10.1371/journal.pone.0203406)

**S2 Fig. KM curves for (A and B) TTP, (C and D) TTD, and (E and F) TTDeath, stratified by (A, C, E) objective response or (B, D, F) lack of objective response.**


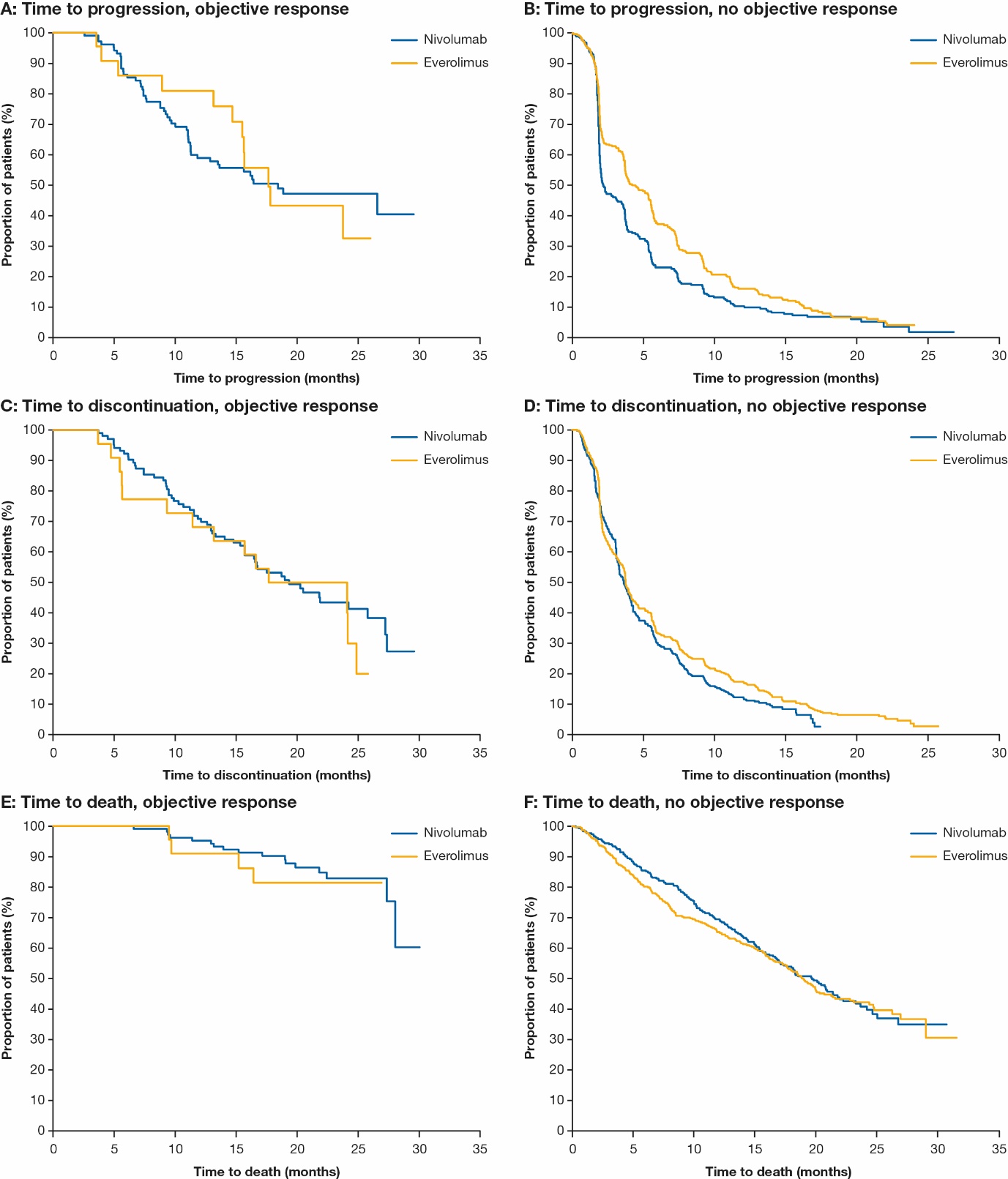

Supplement: S2 Fig — KM curves for (A and B) TTP, (C and D) TTD, and (E and F) TTDeath, stratified by (A, C, E) objective response or (B, D, F) lack of objective response. (DOCX) [file pone.0203406.s004.docx]
